# Microbiological and molecular aspects of periodontitis pathogenesis: an infection-induced inflammatory condition

**DOI:** 10.3389/fcimb.2025.1533658

**Published:** 2025-05-08

**Authors:** Mina Yekani, Masoumeh Dastgir, Samaneh Fattahi, Shahriar Shahi, Solmaz Maleki Dizaj, Mohammad Yousef Memar

**Affiliations:** ^1^ Dental and Periodontal Research Center, Tabriz University of Medical Sciences, Tabriz, Iran; ^2^ Infectious and Tropical Diseases Research Center, Tabriz University of Medical Sciences, Tabriz, Iran; ^3^ Department of Medical Physics, School of Medicine, Tabriz University of Medical Sciences, Tabriz, Iran; ^4^ Students Research Committee, Tabriz University of Medical Sciences, Tabriz, Iran; ^5^ Department of Oral and Maxillofacial Medicine, Faculty of Dentistry, Tabriz University of Medical Sciences, Tabriz, Iran

**Keywords:** periodontitis, biofilm, inflammation, microbiology, treatment

## Abstract

Periodontitis (PD) is the most common oral infectious disease. The primary etiologic cause of the onset and development of PD is dental plaque, which consists of bacterial biofilm domiciled within a complex extracellular mass. In PD patients, there is a progressive breakdown of the periodontal ligament and the alveolar bone. In more advanced stages, tooth loss occurs. The progression of this chronic inflammatory disease involves interactions among numerous microbial pathogens particularly, bacteria, the host’s immune factors, and various environmental factors. Due to persistent infection by periodonto-pathogenic bacteria, there is an impairment of both innate and acquired immunity, leading to tissue destruction. Chronic inflammation in PD may be associated with several systemic diseases, including cardiovascular conditions, respiratory issues, diabetes, neurological diseases, cancer, and adverse pregnancy outcomes. Antibiotic treatment is one of the effective strategies for treating PD cases, although the emergence of some resistant strains may limit the effectiveness some antibiotics. In this review study, we discussed the main bacteria in PD, the interaction with the immune response, the pathogenesis of bacteria in PD and antibiotic treatment. We also outlined the emergence of resistance to antibiotics among these pathogens.

## Introduction

Periodontitis (PD) is a significant public health challenge with a high prevalence and noticeable socio-economic impacts. PD significantly affects patients’ quality of life. It is reported that, over the last decade, the prevalence of PD has reached nearly 60%, which is an increased compare to the same time period in the past (Trindade et al., 2023). PD is a chronic inflammatory condition, primarily caused by the formation of the microbial on dental surfaces, which damages the soft tissue surrounding the teeth. The biofilm consists of a complex community of microorganisms surrounded by an extracellular matrix of biological macromolecules. Biofilm is highly resistant to antimicrobial drugs and host immunity mechanisms. An imbalance in the microbial composition of biofilms, triggers host’s immune response, which leading to a chronic inflammatory condition. Gingivitis is a mild and reversible form of inflammation. If not treated properly, it can progress to PD, a chronic condition that can eventually lead to the entry of bacteria or their byproducts into the bloodstream. This, in turn, triggers the host’s inflammatory response through various mechanisms (Schenkein et al., 2020; Zhang et al., 2022). PD is characterized by damage to the periodontal ligament, pockets formation, and resorption of alveolar bone. These symptoms are induced by the host’s inflammatory response to bacterial biofilms, which express bacterial virulence factors (Preshaw and Bissett, 2019). Cytokines and immune mediators play a crucial role in the progression of PD by regulating inflammatory signals that control local inflammation and tissue destruction (Neurath and Kesting, 2024). The long-lasting presence of plaque on the surface of teeth leads to its migration into the neighboring periodontal tissues and stimulates the infiltration of host immune cells. The progression of PD is often affected by life style factors, such as deficient oral hygiene and smoking, compounded with genetic and disease-related compromise of the body’s immune response (Gao et al., 2018; Jensen et al., 2018; Deo and Deshmukh, 2019). The most common pathogens that caused PD are *Porphyromonas gingivalis*, *Treponema denticola* and *Tannerella forsythia* (Saquib et al., 2021). Understanding the microbiology and pathogenesis of PD, as well as researching treatment options and drug resistance of the causative agents, will greatly improve the effectiveness of treatment. Currently there are few studies that comprehensively outline the microbiological aspects, inflammatory pathways involved in pathogenesis, antibiotic resistance, virulence factors of the causative microbial agents, and alternative treatment options for PD. The aim of the present study was to discuss the main bacteria involved in PD and their interaction of these bacteria with the immune response. We also provided an overview of the pathogenesis of bacteria in periodontitis and antibiotic treatment and emergence of antibiotics resistance. In this literature review, data on microbiology, pathogenesis, microbial resistance, and treatment of PD were gathered from databases including Google Scholar, Scopus, and PubMed. Searches were conducted using keywords such as “PD microbiology,” “PD pathogenesis,” “PD treatment,” and “antimicrobial resistance in PD-causing pathogens.” All English language articles retrieved were independently reviewed by two individuals.

## Pathogenesis of periodontitis

The prolonged presence of plaque on the surface of teeth causes it to migrate into the surrounding periodontal tissues. This migration stimulates the infiltration of host immune cells, particularly polymorphonuclear neutrophils (PMNs), from the blood to the site of infection. It has been proposed that severe forms of PD, which primarily affect young individuals previously categorized as having “aggressive periodontitis” (AgPD) or grade 3 periodontitis (involving resorption of ≥2 mm over five years), may be associated with more unfavorable impacts compared to the more prevalent form known as “chronic periodontitis” (CPD) (Borilova Linhartova et al., 2020; Fuller et al., 2020). AgPD cases are commonly reported in individuals under the age of 30 years and exhibit a rapid progression that poses challenges in treatment (Clark et al., 2017). In contrast, CPD tends to progress at a slower rate. Despite their differences, both AgPD and CPD can present in more severe forms ultimately leading to tooth loss and edentulism (Brito et al., 2018). Although AgPD and CPD share several similarities, there are notable clinical differences between the two, including: (i) age of disease manifestation (i.e.detection), (ii) rate of disease progression, (iii) destructive patterns, (iv) indications of inflammation, and (v) plaque and calculus levels (Armitage and Cullinan, 2010). CPD is characterized by the gradual progression of the disease over time in the absence of appropriate treatment, while AgPD is characterized by rapid attachment loss and bone destruction (Cardoso et al., 2018).

**Figure 1:**
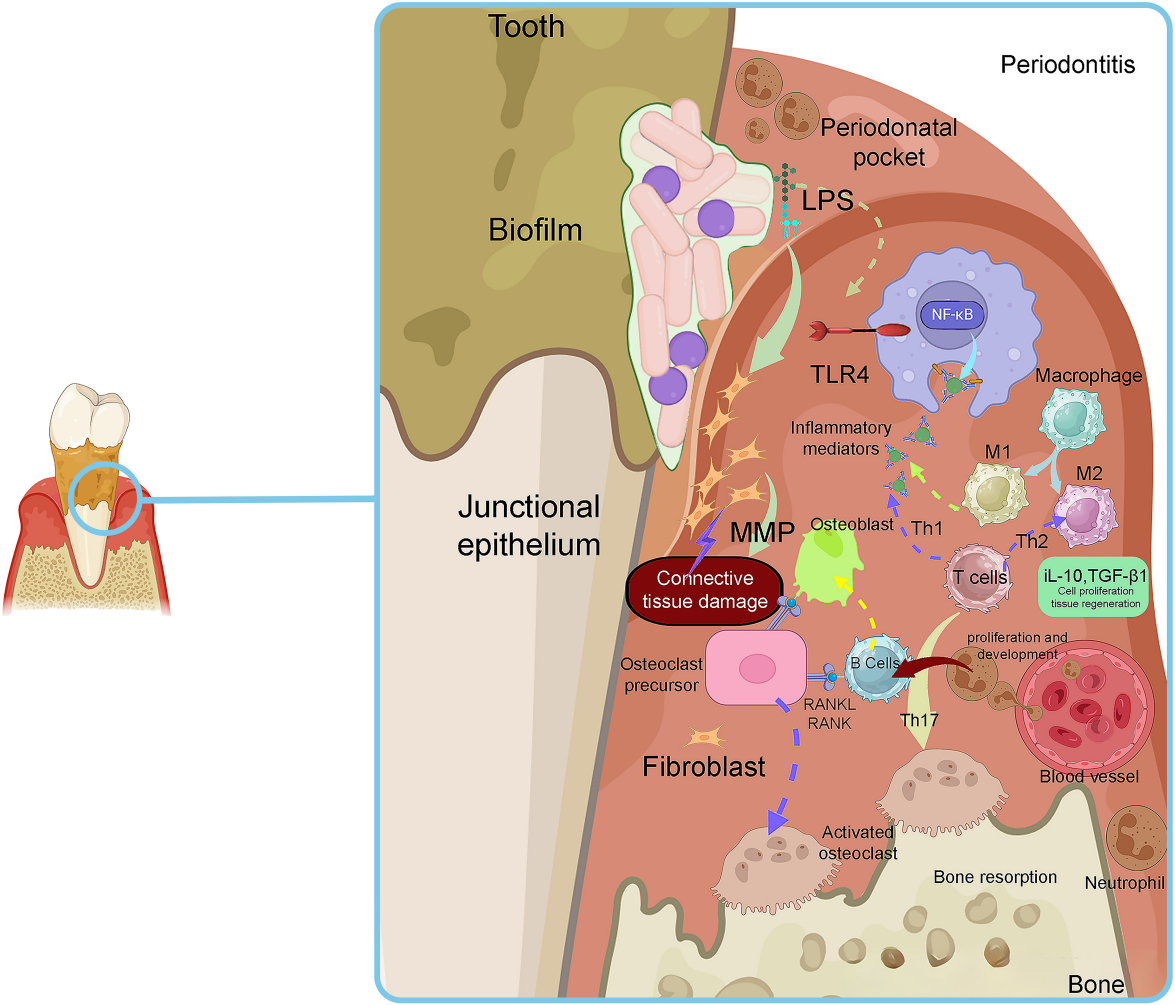
The histopathological aspects of plaque-induced inflammation in PD. Adapted from Sara I Pai et al (Pai et al., 2023).

The primary cause of periodontal inflammation is the microbial biofilms that form on the surface of teeth and expand to the gingival crevice (Larsen and Fiehn, 2017). Bacteria in these biofilms exploit their pathogenicity by causing damage to gingival tissue using direct and/or indirect mechanisms. Direct damage is caused by several bacterial factors that affect the cells and intercellular matrix of the host connective tissue. These bacterial factors generally involve secreted bioactive molecules, such as exoenzymes, toxins, and metabolic end-products (Siqueira and Rôças, 2024). Moreover, some somatic contents of bacteria, such as cell wall contents (peptidoglycan and lipoteichoic acid of Gram-positive bacteria and lipopolysaccharide (LPS) of Gram-negative bacteria), fimbriae, flagella, outer membrane proteins (OMPs), vesicles (OMVs), nucleic acids, and exopolysaccharides, are released into the adjacent tissues and stimulate the host immune mediators (Siqueira and Rôças, 2019). **Table 1** is presents the virulence factors of common bacterial pathogens involved in PD. Bacterial components can trigger inflammatory and non-inflammatory host cells to express immunological factors and mediators, such as cytokines and prostaglandins. These mediators have a stimulating effect on bone tissue resorption, which is a characteristic feature observed in PD lesions (Lamont et al., 2020) (**Figure 1**). The purulent exudate formed in acute apical abscesses is another example of indirect bacterial damage to gingival tissue. The host’s immune response to bacterial antigens originating from the root canal is considered the primary factor in the development of pus associated with abscesses (Karamifar et al., 2020). When periodontal tissues encounter bacterial cell-associated components known as pathogen-associated molecular patterns (PAMPs), certain host cell receptors referred as pattern recognition receptors (PRRs), such as Toll-like receptors (TLRs) recognize the pathogens and trigger the host immune response. TLRs are expressed on the surface of gingival epithelial cells, fibroblasts, dendritic cells (DCs), and macrophages (Becerra−Ruiz et al., 2022). PRRs including TLRs, are capable of recognizing conserved regions of PAMPs such as LPS, peptidoglycans, bacterial DNA, and lipoproteins (Li and Wu, 2021). TLRs play an essential role in triggering innate immunity. They are evolutionarily conserved and provide the first line of defense against microbial pathogens upon entry into host tissues (Kawai et al., 2024). Proinflammatory reactions induced by TLRs rely on the stimulation of the nuclear factor kappa B (NF-κB) and mitogen-activated protein kinase (MAPK) pathways. These pathways coordinate the transcription and synthesis of proinflammatory mediators such as cytokines and chemokines (Collins et al., 2019). Thereafter, non-resident leukocytes, such as PMNs are attracted to the site of infection in response to specific cytokines and chemokines produced (Duan et al., 2022). PMNs are the most infiltrated leukocytes (50%-70%) and serves as an initial defense against the microbial pathogens in dental plaque. PMNs play a critical role in maintaining the healthy condition of periodontal tissue and provide the first-line mechanisms in the innate immune system. PMNs employ various specific mechanisms of action, including degranulation, chemotaxis, phagocytosis, formation of reactive oxygen species (ROSs), and the formation of neutrophil extracellular traps (NETs) (Sczepanik et al., 2020).

**Table 1:** The virulence factors of the most important bacterial pathogens causing periodontitis.

Bacteria	Virulence factors	Role in pathogenesis	Ref.
***Tannerella forsythia***	Proteases (KLIKK)	Degradation of collagen, gelatin, elastin, and casein and contribution to damage the connective tissue at the infected periodontal tissue	(Ksiazek et al., 2015)
	Proteases (PrtH)	Protect the bacteria from destruction by complement system and antimicrobial peptides	
	Dipeptidyl peptidase IV	A serine protease that cleaves X-Pro or X-Ala dipeptide and contribution collagen destruction	(Yost and Duran-Pinedo, 2018)
	Miropin	Inhibition a broad range of target proteases, including neutrophil-derived cathepsin G and elastase	(Eckert et al., 2018)
	Glycosidases	Break down oligosaccharides and proteoglycans in saliva and periodontal tissues, contributing to disease progression	(Philips et al., 2020)
	OxyR protein	Resistance to oxidative stress	(Prucsi et al., 2021)
	Outer Membrane Vesicles (OMVs)	Regulation of stress responses, quorum sensing, horizontal gene transfer, co-aggregation of bacteria and biofilm formation and releasing of proinflammatory and immunoregulatory cytokines	(Srisatjaluk et al., 1999; Chi et al., 2003; Sharpe et al., 2011; Cecil et al., 2016)
	Leucine-rich repeat BspA protein	Attachment and penetration into host cells such as fibronectin and clotting factor and production of proinflammatory mediators	(Sharma, 2010)
	Sialidases (SiaHI and NanH)	SiaHI function is unknown. NanH sialidase adhere to sialylated glycoprotein-coated surfaces and epithelial cells, and it triggers biofilm formation	(Megson et al., 2015)
	S-layer	Modulate host immune responses, adherence, colonization and tissue invasion	(Schäffer and Andrukhov, 2024)
	Type IX secretion system (T9SS)	Role in S-layer formation, motility, and biopolymer degradation and utilization	(Paillat et al., 2023)
	Karilysin	Degrade elastin, fibrinogen and fibronectin, inactivate the antimicrobial peptide, and induce TNF-α expression	(Eckert et al., 2018; Săndulescu et al., 2023)
	High-temperature requirement A (HtrA) protease	Surviving, adaptation to environmental alteration, and tolerance of unfavorable conditions such as high-temperatures (heat-shock response), extreme pH and oxidative and osmotic stress	(Hansen and Hilgenfeld, 2013)
***Porphyromonas gingivalis***	Gingipains arginine-specific gingipain (Rgp A, Rgp B), lysine-specific gingipain (Kgp)	Process of surface-associated proteins and hemagglutinins, attachment, growth, development, and escape of host defense	(Seers et al., 2021)
	Fimbriae (pili); long or major FimA fimbriae, and short or minor Mfa1 fimbriae	Biofilm forming, auto-aggregation, co-aggregation with oral bacteria, adhere to host molecules, and invade to host cell	(Hasegawa and Nagano, 2021)
	Hemolysin	Degrade erythrocyte membrane, causing the release of hemoglobin	(Aleksijević et al., 2022)
	Hemagglutinin	Adhesin to host cells and facilitates the acquisition of heme through erythrocyte	(He et al., 2020)
	Capsule	Induce osteoclast differentiation and alveolar bone loss by inducing Th1 and Th17 immunity cells	
	OMVs	Containing adhesive and proteolytic molecules can easily merge with host cells and transfer their contents directly into the host cell cytosol, spreading virulence factors to various tissues as they travel and disperse	(Zhang et al., 2022)
	LPS	Produces of pro-inflammatory mediators, including TNF-α, IL-1β and nitric oxide (NO), and rises permeability of gingival epithelium, and plays a significant role in alveolar bone resorption	(Gu et al., 2022)
***Treponema denticola***	Dentilisin (chymotrypsin-like protease (CTLP)	Disruption of the extracellular matrix (ECM) and serum proteins, cytotoxic, destroying host tissues, involving in nutrient uptake, bacterial coaggregation, activation of complement proteins, the scaping of the host immune response, the inhibition of the hemostasis system, and cell invasion	(Hernández-Jaimes et al., 2022; Hinson et al., 2022; Yokogawa et al., 2022)
	Dentipain	Resistance to opsonization and phagocytosis and inactivating the dentipain protease domain attenuated the abscess-formation	(Miyai-Murai et al., 2023)
	Major sheath protein (Msp)	Cytotoxic effects, colonizes in host tissues, protects itself from the cytopathic pore-forming activity against epithelial cells, bind to keratin, collagen type 1, fibrinogen, hyaluronic acid, and heparin,degrades extracellular matrix (ECM) and serum proteins	(Hinson et al., 2022; Pisani et al., 2023)
	Motility and Chemotaxis	Motile in a highly viscous environment which is beneficial for its movement through polymicrobial biofilms and allowing better nutrient penetration and waste removal	(Ng et al., 2019)
	Lipooligosaccharide (LOS).	Attach to ECM proteins, mucosal cells, and oral bacteria and potentially enhancing their pro-inflammatory effects	(Visentin et al., 2023)
***Aggregatibacter actinomycetemcomitans***	Leukotoxin (LtxA)	Rapidly degrade white blood cells (WBCs), aiding the bacteria to subvert the host defense	(Kachlany and Vega, 2024)
	Cytolethal distending toxin (Cdt)	Disrupting the host response results in a decrease phagocytic activity, intensify inflammation, and neutolizes the immune defense. CdtB induces irreversible cell cycle arrest and subsequent cell death by triggering the apoptotic pathway in various target cells. CdtB causes DNA damage	(Shenker et al., 2022; Kim et al., 2024)
	Fimbrial adhesion bundle-forming type IVb-like fimbriae	Bind to host tissues and abiotic surfaces	(Inouye et al., 1990; Kachlany et al., 2001; Danforth et al., 2019)
	Nonfimbrial adhesion	Aae and Omp100/ApiA (human oral epithelial cells), extracellular matrix adhesin protein A, EmaA, and Omp100/ApiA(collagen)	(Asakawa et al., 2003; Rose et al., 2003; Mintz, 2004; Yue et al., 2007; Tang et al., 2008)
	Heat shock protein 60 (HSP60)	Survive the microorganisms under stress conditions (temperature, pH, redox potential, oxidative stress, etc.)	(Yamamoto and Eguchi, 2020)
***Fusobacterium nucleatum***	Adhesins (Aid1, CmpA, Fap2, FomA, FadA and RadD)	FadA increases fusobacterial adherence to biofilms by connecting various Gram-positive initial colonizers. RadD aids to attachment to both bacteria and the yeast *Candida albicans*. Fap2 binds to the Gal-GalNAc polysaccharide produced by CRC cells, inducing lymphocyte death, or to immunoglobulins and ITIMs, suppressing immune cell activity. Aid1 and CmpA are also involved in these interactions.	(Pignatelli et al., 2023) (Kaplan et al., 2010),
	Fn-Dps (DNA hunger/stationary phase protective proteins)	Assisting to its survival in macrophages and damage erythrocyte.	(Wu et al., 2023) (Li et al., 2023),
	LPS	Activation of the immune response.	(Garcia-Vello et al., 2021)
	OMVs	Harboring toxic bacterial factors are continuously released during *F. nucleatum* growth and modulate the inflammatory response	(Chen et al., 2022)
	Butyrate Production	induce the proliferation of colonic epithelial and immunosuppressive cells, which subsequently promotes tumor development	(Brennan et al., 2021) (Ye et al., 2024),
***Veillonella parvula***	LPS	Activate the complement proteins	(Nygren et al., 1979)
	H_2_S production	Toxic to host cells	(Rôças and Siqueira, 2006)
***Prevotella***	Adhesion 29KDa OMPs Adpb.	High affinity to laminin and fibronectin which binds to collagen	(Yu et al., 2006; Marre et al., 2020)
	Hemolysin	Provide a heme for proliferation by degrading red blood cells	(Guan et al., 2006)
	Hemagglutinin	Attainment of heme from erythrocytes	(Naito et al., 2022)
	Proteolytic activity	Development and progression of infection	(Yanagisawa et al., 2006)

PMNs are central player in inflammation as they migrate to infected tissues to eliminate invading microorganisms. While PMNs in the gingival crevice can interact with bacterial biofilms on tooth surfaces, they are unable to completely eliminate the entire population of periodontal microorganisms (Alfakry et al., 2016). Whether there is an overabundance or a deficiency of activated PMNs, their role in causing periodontitis remains crucial (Hajishengallis et al., 2015). On the contrary, excessive activation of PMNs can lead to elevated levels of toxic substances and ROS, which characterize localized AgPD (Hasturk and Kantarci, 2015). ROS production can activate granular proteases, leading to the formation of NETs, which are networks composed of extracellular fibers of DNA that restrict invading microorganisms. Moreover, ROS can increase the permeability of bacterial membranes, causing damage to their DNA, proteins, and cell envelope. ROS formation serves as a potent antimicrobial defense mechanism and plays a crucial role in innate immunity against pathogens (Brings et al., 2020; Veenith et al., 2022).

In various types of PD, PMNs can either contribute to antimicrobial defense or tissue damage (Ramadan et al., 2020). PMNs also play a role in periodontal tissue damage by releasing degradative enzymes, such as matrix metalloproteinases (MMPs). MMPs are a class of enzymes responsible for breaking down the ECM contents, including collagen and elastin. In PD, the synthesis of MMPs is increased, primarily by immune cells and resident cells in the periodontal tissues. The overactivity of MMPs leads to the degradation of connective tissue fibers and contributes to alveolar bone loss in PD. PMNs can directly promote osteoclastic bone resorption by increasing the expression of membrane-bound receptor activator of nuclear factor-κB ligand (RANKL), a critical osteoclastogenic cytokine. PMNs also have the ability to release collagenase, which plays an essential role in bone resorption (Hajishengallis et al., 2016). However, PMNs typically do not express soluble RANKL (Chakravarti et al., 2009), and are only capable of mediating periodontal bone resorption when they are adjacent to the bone tissue. During inflammatory conditions, osteoclast progenitors respond to several mediators produced by induced CD4^+^ T cells including RANKL, TNF-α, and interleukins (IL)-17. Among CD4^+^ T cells, Th17 cells have been demonstrated to promote osteoclast differentiation. Th17 cells increase expression of IL-17 in inflammatory conditions, which is associated with increased bone destruction and osteoclastogenesis by upregulating RANK in osteoclast progenitors and increasing RANKL expression in osteoblasts (Madel et al., 2019). Osteoprotegerin (OPG) is a member of the TNF receptor superfamily, and acts as a soluble decoy receptor. It inhibits the interaction of RANKL with its functional receptor on osteoclast precursors (Liu and Zhang, 2015). The expression of RANKL and OPG may also be affected by amelogenin, which regulates odontoclast formation (Galler et al., 2021). A recent study showed that bone resorption is a common feature of PD. This process is intensified by increased RANKL expression and simultaneous downregulation of OPG, leading to an elevated RANKL/OPG ratio and subsequent activation of osteoclasts (Abdullameer and Abdulkareem, 2023).

Approximately one third of the variance observed in the frequency of periodontitis can be attributed to genetic factors. This heritability index has been consistent across different populations studied and tends to increase with the severity of PD (Nibali et al., 2019). Genetic factors play a role in regulating inflammatory responses within affected tissues and the damage induced in the alveolar bone (Tettamanti et al., 2017). Numerous scientific studies have investigated the influence of genes and their variants (polymorphisms) on host responses in periodontitis. Genetic polymorphisms can lead to changes in the encoded proteins or their expression, potentially altering innate and adaptive immune responses, and influencing disease outcomes. Interestingly, certain genetic polymorphisms may also provide protection against disease (Laine et al., 2012). Polymorphisms, defined as genetic variants occurring in at least 1% of a population, originate from mutations. Approximately 90% of polymorphisms are Single Nucleotide Polymorphisms (SNPs), in which a single nucleotide base is replaced by another. While most SNPs in genes do not alter the protein produced, they can still affect the gene’s function. Given that all forms of PD are associated with bacterial infections, it remains challenging to delineate the relative contributions of genetic and environmental factors to these complex disorders. Specific genetic polymorphisms, such as those in Interleukin-1 (IL-1), IL-6, IL-10, Fcγ receptors (FccR), Vitamin D receptor (VDR), and TNF-α genes, have been shown to be connected with periodontitis (Laine et al., 2012; Sayad et al., 2020). Similarly, polymorphisms in matrix metalloproteinase (MMP) genes may influence the expression or activity of MMPs, potentially increasing susceptibility to periodontal conditions (Li et al., 2016). Some studies have identified associations between the expression of RANKL/RANK/OPG triad elements and PD (Cochran, 2008). Although no genetic polymorphism in the RANKL/RANK/OPG triad genes has been confirmed as a risk factor for root resorption, further research is needed to explore this possibility. Studies have shown that overexpression of RANK in monocyte/macrophage lineage cells and gingival epithelial cells in mice led to decreased alveolar bone height and an increased number of TRAP-positive cells in the alveolar bone. This suggests excessive osteoclast activity and accelerated bone resorption, possibly due to inflammation in the gingival epithelium, which occurs prior to any detectable bone loss (Sojod et al., 2017).

## Dental plaque formation

**Figure 2:**
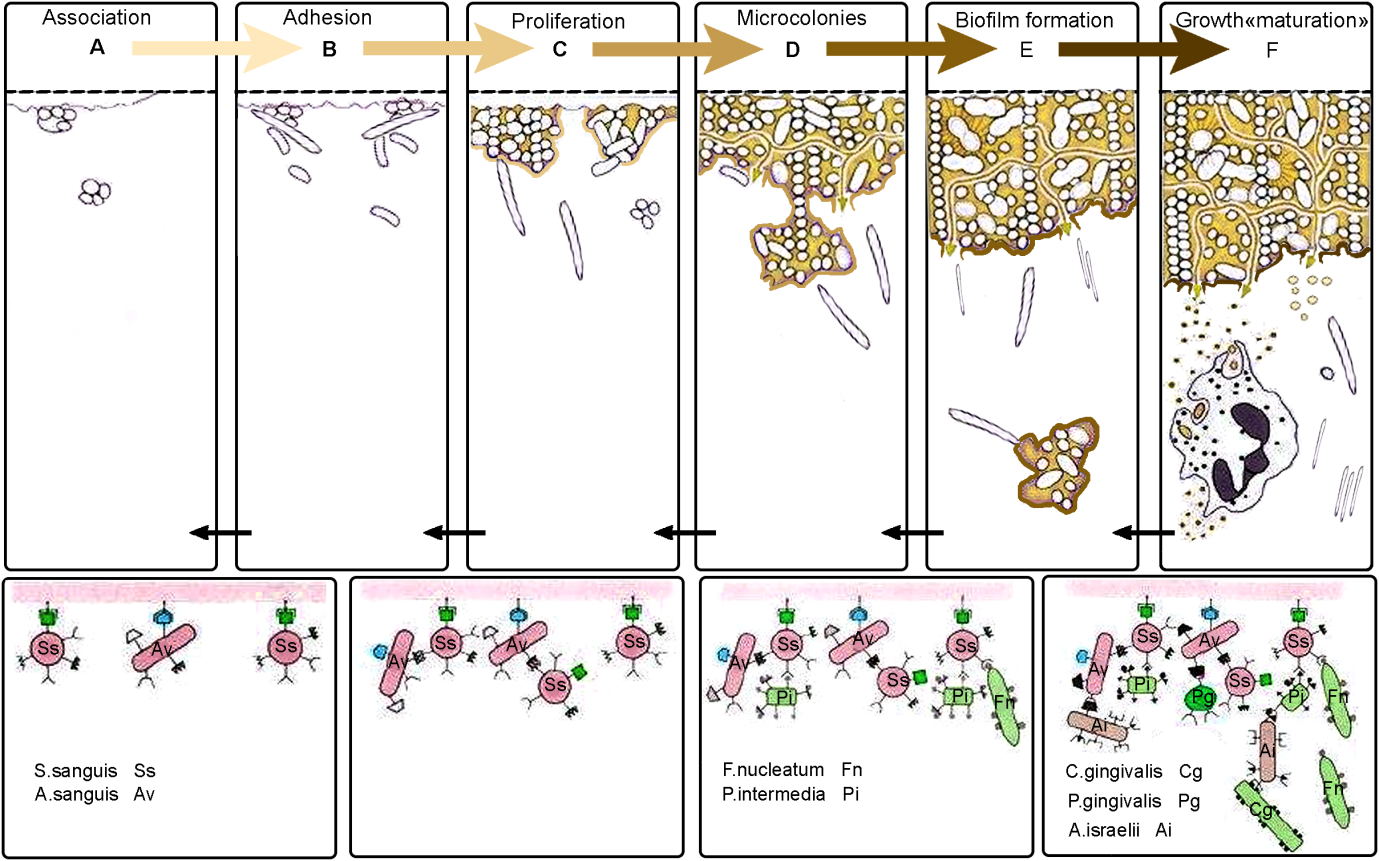
Biofilm formation stages on teeth surface. **(A)** Association: Free-floating bacteria come into contact with the dental surface, which is coated with an acquired pellicle. This pellicle is a layer made up of proteins and glycoproteins that come from saliva. **(B)** Adhesion: Primary colonizing microorganisms firmly attach to the pellicle using specific adhesion molecules. **(C)** Proliferation: These bacteria multiply and begin to form microcolonies, producing extracellular polymeric substances (EPS) that strengthen and stabilize the developing biofilm structure. **(D)** Microcolonies: Facultative anaerobic and anaerobic bacteria, such as *Fusobacterium nucleatum* and *Prevotella* intermedia, integrate into the microbial community. This integration allows for interactions with more pathogenic bacteria, like *Porphyromonas gingivalis*. **(E)** Biofilm Formation: As the biofilm continues to develop, it becomes increasingly complex and denser. The structural organization of the biofilm allows for the formation of channels that regulate nutrient diffusion and waste removal within the microbial community. **(F)** Growth "maturation": Some bacteria, particularly planktonic forms, detach from the biofilm. These detached cells become free-floating within the fluid environment of the oral cavity. Once separated, these planktonic forms can reattach to the acquired pellicle that forms on freshly cleaned tooth surfaces. This initiates a new cycle, continuing through the phases of association, adhesion, proliferation, microcolony, and biofilm maturation. This cycle repeats, leading to continuous biofilm development.

Dental plaque, also known as microbial plaque, oral biofilm, or dental biofilm, is a complex and highly organized community of microorganisms. Biofilm forms on the surfaces of teeth and is embedded in a matrix of polymers from both host and bacterial origins. Oral biofilms play a crucial role in the development of a variety of diseases in the oral cavity and throat, including dental caries, PD, endodontic infections, tonsillitis, and alveolitis (Colombo et al., 2015). Biofilm formation begins with the initial adhesion of single free floating, planktonic cells to a surface (Sauer et al., 2022) (**Figure 2**). Loose, long-range physicochemical interactions between the microbial cell surfaces and the pellicle-coated tooth enable reversible adhesion through van der Waals forces and hydrophobic interactions. This is followed by more robust and closer interactions between ligands on the surface of the early colonizers and matching receptors in the pellicle, ultimately leading to permanent attachment through their pili (see **Figure 2**). Thus, the acquired pellicle, largely derived from the host, actively guides the pattern of initial microbial colonization (Barnier et al., 2021; Darveau and Curtis, 2021). Bacterial biofilm is composed of bacteria that are enclosed in a polymeric matrix that they produce (Bîrluţiu et al., 2017). During this process, a self-generated matrix of extracellular polymeric substance (EPS) forms a tough barrier, shielding the bacteria from external stress (Das, 2022). The accumulation of small bacterial clusters and layers leads to the formation of plaque biofilm. In PD, mature biofilm is characterized by matrix macromolecules such as extracellular DNA (exDNA). This exDNA exists in two forms within the matrix: free DNA and extracellular DNA traps (ETs). Regardless of its origin, exDNA is essential for initiating biofilm formation and maintaining its three-dimensional structure (Wei et al., 2024). Microbial communities within the biofilm engage in a series of physical, metabolic, and molecular interactions that can influence antibiotic resistance and pathogenicity. Studies have shown that microbial cells embedded in the biofilm are 10–1000 times more resistant to antibiotics compared to planktonic cells (Digel et al., 2020). Bacterial attachment to biomaterial surfaces involves a variety of physicochemical interactions and biological processes, with mechanisms that are specific to either the bacteria or the substrate (Kreve and Dos Reis, 2021). When saliva contacts the teeth, proteins from the saliva stick to the tooth surface, creating an acquired salivary pellicle. This pellicle covers all tooth surfaces in the mouth and serves as a link between the dental hard tissue and the oral environment (Chawhuaveang et al., 2021). The pellicle is primarily composed of proteins, amino acids, fatty acids, glycoproteins, carbohydrates, lipids, and other compounds present in saliva. It also contains microorganisms like bacteria and fungi. This layer acts as a lubricant and provides protective properties for the teeth (Enax et al., 2023). The attachment of bacteria to the surfaces of teeth during dental plaque formation occurs through a hydrophobic interaction. This interaction involves a phenylalanine side chain of bacterial surface molecules and a leucine side chain of a salivary glycoprotein in the formed pellicle. Additionally, calcium bridging (takes place, which is the results of an electrostatic attraction) between a negatively charged carboxyl group of a bacterial protein and a positively charged calcium ion.

Furthermore, dietary sucrose is transformed by bacterial glucosyltransferase into glucan. Glucan contains multiple functional residues that can interact with amino acid side chains like serine, tyrosine, and threonine. Bacterial fimbriae play a role in providing the terminal adhesin portion necessary to bind to a sugar component of a salivary glycoprotein in the acquired pellicle (Digel et al., 2020).

Streptococci compete for adhesion sites on the saliva-covered surface of teeth and can produce antimicrobial substances. *S. mutans* can dominate oral biofilms, leading to the development of dental caries (Moschioni et al., 2010). *S. mutans* is the primary early colonizing bacteria responsible for dental caries because of its ability to recognize salivary pellicle receptors. *S. mutans* antigen I/II (Ag I/II) is a key factor in this process, playing a vital role in adhering to the tooth surface and in microbial co-aggregation during the initial stages of biofilm formation (Rivera-Quiroga et al., 2020). Oral bacteria that are unable to adhere to surfaces are carried into the digestive system through salivary flow. However, many oral bacteria have developed mechanisms to attach to solid surfaces including teeth covered with salivary films or other bacteria already attached to these surfaces (co-aggregation), as well as epithelial layers (Kolenbrander et al., 2010). In addition to Ag I/II (also known as SpaP, Pac, P1), *S. mutans* also produces glycosyltransferases (Gtfs), various glucan-binding proteins (Gbps), and collagen-binding proteins, which play a role in coordinating the plaque formation (Matsumoto-Nakano, 2018). *S. mutans* biofilm formation is a complex process involving interactions between proteins and bacteria. It begins with the adhesion of a single cell, followed by accumulation, microcolony formation, and eventually developing into a mature biofilm (Krzyściak et al., 2014). Adherence to host tissues is a crucial step in the pathogenic process, typically facilitated by bacterial surface-exposed proteins. In *S. mutans*, adhesion mechanisms include both sucrose-dependent (requiring Gtfs) and Ag I/II -dependent pathways (Scharnow et al., 2019).

Without sucrose, *S. mutans* produces key adhesins like Ag I/II, which specifically attach to a glycoprotein known as salivary agglutinin (SAG) (Lamont et al., 1991; Mitchell, 2003). It has been suggested that this also plays a role in bacterial adhesion to teeth (Jakubovics et al., 2005), and biofilm formation. Various oral bacteria have the LuxS/AI-2 quorum sensing (QS) system. LuxS contributes to biofilm formation, regulates acid and oxidative stress tolerance, and controls the production of the lantibiotic mutacin I. Additionally, AI-2-like signaling molecules can influence carbohydrate metabolism and biofilm matrix composition (Wang et al., 2017).

## Microbiology of periodontitis

It is crucial to highlight that PD seems to be initiated by a relatively small group of periodontal pathogens within the intricate dental biofilm (Popova et al., 2013). The currently identified periodontal pathogens constitute only a small fraction of the approximately 600 bacterial species that can colonize dental surfaces both above and below the gingival margin, as well as the oral mucous membranes. Clinical and experimental evidence confirm that certain bacterial strains in the periodontal environment can cause inflammation of the gingival tissue and lead to bone destruction. These strains are known as periodontal pathogens (Paster et al., 2006). Over the past three decades, there has become widely believed that PD is polymicrobial infections (Armitage, 2004; Taba et al., 2005). Only a small percentage of the bacteria in dental biofilm are considered pathogenic for periodontal tissues. It is well established that the majority of periodontal pathogens are anaerobes. However, the biofilm can also contain facultative aerobes, capnophiles, and microaerophiles. Their numbers vary based on the conditions within the established biofilm and periodontal pocket. Most periodontal pathogens are the primary agents responsible for actual PD. Certain bacterial species in the periodontal environment, such as Actinomyces, Streptococcus, and Staphylococcus spp., which are part of the commensal flora, can cause opportunistic infections if the ecosystem is disturbed. Detection of Enterobacteria, viruses, and Saccharomyces spp. in periodontal pockets may indicate a superinfection linked to a destructive periodontal process. Some studies have focused on identifying the subgingival flora most characteristic of specific PD. While findings have linked certain periodonto-pathogens to specific periodontal conditions, there is still no definitive evidence that particular bacterial species are unique to different types of PD. Even in small amounts, these bacteria can cause damage to periodontal structures (Socransky et al., 1998; Mombelli et al., 2002).

Microorganisms can cause disease directly by invading host tissues or indirectly through bacterial factors and toxins. In order to be considered a pathogen associated with PD, a microorganism must possess several key characteristics including:

- The microorganism must be present in higher numbers at infection sites compared to healthy tissues.
- Eliminating the microorganism should be associated with inhibiting the progression of infection.
- The microorganism should express virulence factors that are relevant to the infection process and be capable of eliciting a host immune response.
- *In vivo* pathogenicity assays should indicate the potential of microorganisms to progress PD (Kesic et al., 2008; Posch et al., 2012).

**Figure 3:**
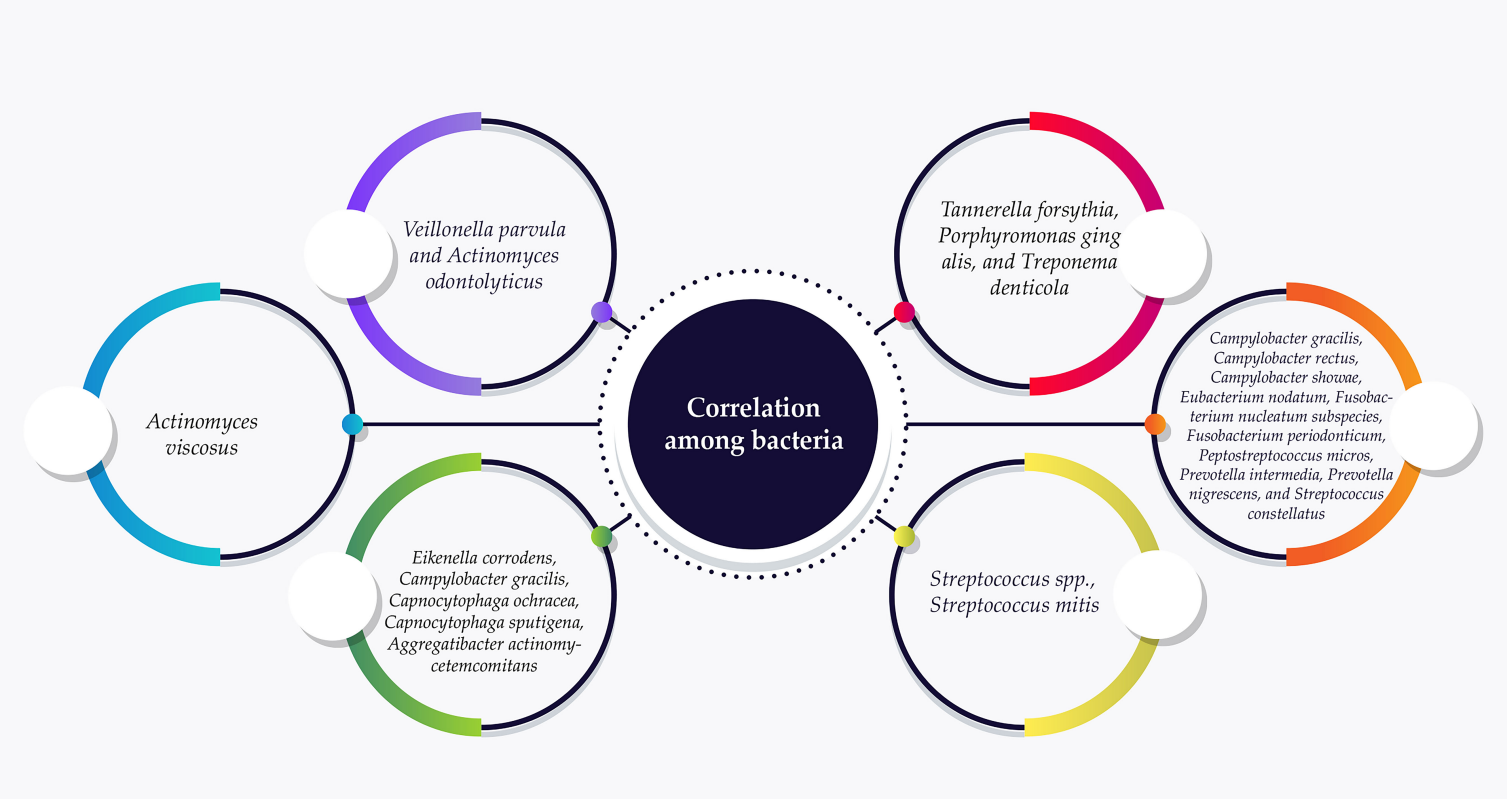
Endodontic-periodontic bacteria commonly assigned in the six complexes according to their association with PD progression. The red complex bacteria are frequently associated with PD and are often detected in deeper spaces near to the epithelial layers of the periodontal pocket.

Studies on endodontic-periodontic bacteria commonly refer to the six complexes initially described by Socranski et al. These complexes are color-coded as blue, green, yellow, purple, orange, and red (Socransky et al., 1998) (**Figure 3**). The red complex bacteria are commonly linked to PD and are often found together in dental plaque, especially in deeper areas near to the epithelial layers of the periodontal pocket. This is largely due to interactions, co-aggregation, and metabolic dependencies among these three bacterial species (Nayak et al., 2018).

The presence of the red complex is closely linked to advanced periodontal lesions, which are characterized by deeper pockets and bleeding (Silva and Cascales, 2021). Additionally, the prevalence and quantity of the red complex increase as the depth of these pockets increases (Mohanty et al., 2019). Next-generation sequencing (NGS) technologies have revealed a wider range of diversity within the oral microbiome and have shown an intricate relationship between microbiome composition and periodontal disease states. This includes a correlation between increased microbial diversity and pocket depth. Recent studies have discovered new microbial connections with PD, emphasizing genera such as *Schwartzia* and *Aerococcus* as linked to the condition. These findings indicate that the microbial environment of PD is more intricate than previously believed (Sisk-Hackworth et al., 2021). A recently identified bacterium from the Bacteriodetes phylum, known as Candidatus Bacteroides periocalifornicus (CBP), has been discovered in dental plaque and shows a strong association with the pathogenic “red complex” found in deep periodontal pockets. CBP is commonly found in the oral cavities of both healthy and diseased individuals but is not detected in gut or skin samples. The abundance of CBP increases with pocket depth and it coexists with *F. nucleatum*, *T. denticola*, and *P. gingivalis*. Its presence is closely linked to members of the red complex rather than healthy commensals, suggesting that CBP could be a new candidate addition to the symbiotic and pathogenic red complex (Torres et al., 2019).

The blue, yellow, green, and purple complexes consist of bacterial species typically associated with a healthy periodontal environment (Carrouel et al., 2016).

Despite the significant focus on the composition of the human microbiome in recent years, the exact mechanisms by which these microbial communities influence disease and maintain health remain largely unknown. Nonetheless, recent studies have revealed that several chronic conditions affecting the mouth and gastrointestinal tract are linked to changes in the microbiome, known as “dysbiosis.” Dysbiosis refers to a harmful shift in the relative abundances and individual components of the microbiome, which vary during healthy states. This shift leads to major dysbiosis-related diseases in humans, including periodontitis, irritable bowel syndrome, chronic vaginosis, and others. Among these, periodontal disease is a prominent dysbiotic condition due to the diverse genera present in both healthy and periodontal microbiomes (Sudhakara et al., 2018).

Oral microbial dysbiosis is a significant factor in the development of oral diseases, including dental caries and periodontal conditions (Hajishengallis, 2015).

This parasitic or pathogenic state, where microbes cause disease in the host, is known as “dysbiosis “or an “unbalanced microbiome” Inflammation plays a key role in altering the microbial community, leading to a continuous cycle of dysbiosis, immune response, and tissue breakdown. The environmental conditions within inflamed periodontal pockets—such as low oxygen levels, enriched nutrients from host protein breakdown, and increased gingival fluid volume—along with synergistic microbial interactions, create a favorable environment for inflammophilic, anaerobic, proteolytic, and fastidious organisms (Kawamoto et al., 2021).

According to Peterson et al., dysbiosis can be identified through three distinct, yet not mutually exclusive, scenarios that may occur simultaneously: i) a general loss of microbial diversity; ii) the loss of beneficial microbes; and iii) the expansion of pathogenic microbes (Petersen and Round, 2014; Van Dyke et al., 2020) These changes provoke an exaggerated inflammatory response in the host via virulence factors, resulting in tissue destruction (Hajishengallis and Lamont, 2012).

In addition to bacteria, the oral mucosal tissues also harbor to various other microorganisms, including archaea, fungi, and viruses. However, research on the relationship between PD and systemic diseases tends to focus primarily on the bacterial component of the microbiome (Unniachan et al., 2020). *Candida albicans* is the predominant fungal species observed in both healthy and infected oral cavities, accounting for over 80% of oral fungal isolates (Talapko et al., 2021). It continues to be the leading fungal pathogen identified in individuals with PD (Jabri et al., 2021). Studies have shown that *C. albicans* can promote the invasion of *P. gingivalis* into human gingival epithelial and fibroblast cells *in vitro* (Tamai et al., 2011). This indicates that C.albicans may contribute to the development or worsening of periodontal disease. The hyphal-specific adhesin Als3, found on the surface of the fungal, is believed be essential for its interaction with *P. gingivalis*. Additionally, pretreating gingival epithelial cells and fibroblasts with *C. albicans* has been demonstrated to increase the invasion of *P. gingivalis* (Montelongo-Jauregui and Lopez-Ribot, 2018). Previous research has investigated the role of Candida species in dental caries, revealing that *C. albicans* significantly contributes to biofilm formation and accumulation (Falsetta et al., 2014; Sztajer et al., 2014). A synergistic relationship between *C. albicans* and oral bacteria enhances the virulence of polymicrobial biofilms, thereby increasing the resistance of fungal cells to antimicrobial agents and environmental stresses (Bartnicka et al., 2019). Specifically in the context of periodontitis, oxygen consumption by *C. albicans* appears to create an oxygen-deficient environment that facilitates the growth of *P. gingivalis* and promotes its ability to invade host cells (Jabri et al., 2022). The pathogenicity of *C. albicans* is linked to its ability to adhere to various surfaces, form hyphae, produce hydrolytic enzymes, invade host tissues, and trigger inflammatory responses (Cavalcanti et al., 2016).

Viruses are recognized as important contributors to the onset and progression of periodontitis. Among them, herpesviruses—including herpes simplex virus-1 (HSV-1), Epstein–Barr virus (EBV), and cytomegalovirus (CMV)—either individually or in combination with other subgingival pathogens, play a pivotal role in both the initiation and progression of the disease (Abooj and Varma, 2021). Herpesvirus infections have the potential to promote the proliferation of bacterial pathogens within periodontal tissues. Macrophages infected with cytomegalovirus or Epstein–Barr virus exhibit a diminished immune response when confronted with periodontal bacteria. Furthermore, proteins produced by herpesviruses on the surface of infected cells can create new binding sites that facilitate the attachment and growth of periodontopathic bacteria (Slots, 2015).

Within the oral microbiome, archaea represent a minor fraction, predominantly consisting of methanogenic phylotypes—strict anaerobes that produce methane. Notable species include *Methanobrevibacter massiliense* (*M. massiliense*), *Methanobrevibacter smithii* (*M. smithii*), and *Methanobrevibacter oralis* (*M. oralis*), which have been linked to periodontitis (Mosaddad et al., 2019; Pilliol et al., 2024). Methanogens have been identified in subgingival samples collected from patients suffering from periodontitis, peri-implantitis, and infected root canals. They have also been detected in saliva specimens from these individuals (Sogodogo et al., 2019). *M. oralis* is the most common archaeal phylotype found in the subgingival biofilm of both healthy individuals and those with aggressive periodontitis, indicating that it may be a regular component of the oral microbiota. While Archaea are generally less abundant and diverse in subgingival regions, the higher levels of methanogens seen in individuals with generalized aggressive periodontitis (GAgP) compared to periodontally healthy (PH) individuals suggest their role in the ecological changes of the microbiota associated with aggressive periodontitis (Matarazzo et al., 2011).

### Tannerella forsythia

*T. forsythia* is an anaerobic Gram-negative bacterium belonging to the *Porphyromonadaceae* family (Posch et al., 2012). It was first discovered in the subgingival periodontal pocket (Miao et al., 2024). *T. forsythia* is non-motile and has a filamentous cell morphology (Assandri et al., 2023). *T. forsythia* employs a highly effective strategy similar to “biological warfare” to enhance its pathogenicity. Specifically, its virulence factors are transported as virulent cargo using outer membrane vesicles (OMVs). *T. forsythia* possess two glycoproteins that play a crucial role in its pathogenicity (Friedrich et al., 2015). *T. forsythia* expresses various important virulence factors, including the leucine-rich repeat BspA protein, sialidases, surface (S)-layer glycoproteins, and dipeptidyl aminopeptidase IV (Table-1) (Sharma, 2020). S-layer glycosylation in *T. forsythia* may be associated with reducing Th17 and innate PMN responses, thereby enhancing the pathogen’s survival within the host. *T. forsythia* also capitalizes on TLR-2-mediated Th_2_ responses to thrive in its primary ecological niche within periodontal pockets. This adaptation ultimately leads to alveolar bone resorption and expansion of its habitat (Settem et al., 2013). Sakakibara et al. described that the glycosylated S-layer of *T. forsythia* contributes to the binding and invasion of human epithelial cell-like gingival carcinoma cells (Ca9–22) and KB cells. Therefore, the S-layer of *T. forsythia* plays a crucial role in the initial stages of PD (Sakakibara et al., 2007). *T. forsythia* lacks the ability to metabolize sugars. It relies on peptides that are degraded by trypsin-like proteases which are involved in the breakdown of smaller peptides but do not play a central role in bacterial virulence, and PrtH cysteine-like proteases (Sharma, 2010). *T. forsythia* expresses a family of six multidomain proteases known as KLIKK proteases, which include three serine proteases (mirolase, miropsin-1, and miropsin-2) and three metalloproteases (karilysin, mirolysin, and forsilysin) (Zak et al., 2021). Karilysin has been found to act as a sheddase, facilitating the release of TNF-α from cell surfaces, and also to cleave and inactivate the antimicrobial peptide LL-37 (Skottrup et al., 2023). Increased levels of *T. forsythia* have been observed in dental plaque samples from post-menopausal women who are obese (Amano et al., 2014), and the bacteria have also been isolated from patients with type 2 diabetes (Miranda et al., 2017). *T. forsythia* is also associated with systemic diseases such as cardiovascular diseases (Carra et al., 2023), and arthritis (Martínez-Rivera et al., 2017). A recent case control prospective study suggested that *T. forsythia* is associated with the development of esophageal cancers (Peters et al., 2017), and lung abscesses (Lv et al., 2023).

### Porphyromonas gingivalis

*P. gingivalis* is a black-pigmented, Gram-negative, rod-shaped, immotile, obligate anaerobe and member of the phylum Bacteroidetes. *P. gingivalis* uses of protein degradation products for providing metabolic energy, heme and vitamin K for its growth (Reyes, 2021; Tanaka et al., 2022). The primary habitat of *P. gingivalis* is the subgingival crevice, but it can develop into a periodontal pocket during PD. Although *P. gingivalis* is native to the human oral cavity, it is detectable in only a small percentage of periodontally healthy individuals (Griffen et al., 1998). The most known *P. gingivalis* virulence factors are fimbriae, hemolysin, hemagglutinins, capsule, OMVs, LPS, and gingipains (Aleksijević et al., 2022). *P. gingivalis* can infiltrate connective tissues by breaking down the epithelial layers and moving between cells, facilitating the spread of the bacteria and may leading to its entry into the bloodstream (de Jongh et al., 2023). The invasiveness of *P. gingivalis* relies on fimbriae. A FimA mutant exhibited a reduced ability to invade gingival epithelial and fibroblast cells. After attachment to gingival sulcular epithelial cells, *P. gingivalis* enters cells and induces remodeling of the actin and tubulin cytoskeleton. Fallowing intracellular proliferation, *P. gingivalis* invades the epithelial cells through actin-based cytoskeletal rearrangements, mediated by FimA interactions with surface epithelial β1 integrins (Khammissa et al., 2018; Chen et al., 2023). *P. gingivalis* produces two types of fimbriae, including FimA and Mfa1, which are essential for the bacteria ability to attach to oral Streptococcus spp., other bacteria in dental biofilms, salivary peptides, and host cells. These fimbriae play a vital role in infections and survival within in the host (Kloppsteck et al., 2016).

Remarkably, both fimbriae of *P. gingivalis* are involved in the invasion of dendritic cells and the induction of adaptive host immune responses (Shahoumi et al., 2023). Gingipains, a group of cysteine proteinases, play a determining role in the pathogenicity of *P. gingivalis* in PD (Xu et al., 2020). They are primarily secreted by a type IX secretion system (T9SS) expressed on the outer membranes and OMVs of most *P. gingivalis* strains or occasionally released into the extracellular milieu as soluble proteins by some *P. gingivalis* strains (HG66) (Ciaston et al., 2022). So far, three types of gingipains have been described in *P. gingivalis* including lysine-specific gingipain (Kgp), arginine-specific gingipain A (RgpA), and arginine-specific gingipain B (RgpB). In addition to their function as proteolytic enzymes for breaking down proteinaceous nutrients to support bacterial growth, gingipains also play an essential role in processing fimbria proteins and in bacterial adhesion and colonization of the host (Singh and Singh, 2022). Gingipains produced by *P. gingivalis* break down C3 into C3a and cleave C5 into the bioactive C5a of the complement system. This process increases proinflammatory cytokines, suppresses the caspase 11-dependent non-canonical inflammasome pathway, and inhibits cell apoptosis, allowing *P. gingivalis* to utilize host cells for its survival (Chopra et al., 2020). The trypsin-like activity of gingipains enables *P. gingivalis* to cleave a variety of host proteins including immunity mediators, blood proteins, ECM proteins, and host cell surface proteins (Li and Collyer, 2011).

The capsule of *P. gingivalis* enhances its pathogenicity by allowing it to evade immune cell recognition and increasing its tolerance to phagocytosis (Sharaf and Hijazi, 2022). *P. gingivalis* primarily acquires heme through mechanisms involving hemagglutinin, hemolysin, and gingipains (Śmiga et al., 2023).

The association of *P. gingivalis* has been described with the development of various gastrointestinal cancers (Tan et al., 2022), pancreatic cancer (Gnanasekaran et al., 2020), atherosclerosis (Zhang et al., 2021), Alzheimer’s disease (Ryder, 2020), rheumatoid arthritis (Li et al., 2022), bacterial vaginosis (Fiorillo et al., 2019), preterm birth (Udagawa et al., 2018), respiratory tract infections (Bregaint et al., 2022), and type 2 diabetes (Tian et al., 2020).

### Treponema denticola

*Treponema denticola* is a Gram-negative, spirochete and anaerobic bacterium commonly detected in CP lesions, often along with *P. gingivalis* and *T. forsythia* (Dashper et al., 2019). The principal virulence determinants of *T. denticola* are found within the toxin–antitoxin (TA) system, specifically transposases (Kuboniwa et al., 2012). These include outer sheath proteins, particularly the major outer sheath protein (MOSP) (Puthenveetil et al., 2017), trypsin-like protease activity (Usui et al., 2022), lipoproteins (Goetting-Minesky et al., 2021), and dentilisin (Ganther et al., 2021). The motility of *T. denticola* plays a crucial role in its ability to penetrate oral epithelial cell layers and is closely tied to its virulence. Like to other spirochetes, the flagella in *T. denticola* are internal and located between the protoplasmic cylinder and the outer sheath. This internal positioning enables the flagella to rotate the cell, facilitating motility. The internal flagella motility helps spirochetes penetrate more viscous layers more effectively than microorganisms with external flagella (Nakamura et al., 2006). This ability plays a crucial role in the colonization of *T. denticola* through biofilm formation (Ng et al., 2019). *T. denticola* activates TLR2/MyD88, leading to the nuclear translocation of the transcription factor Sp1, which is a key regulator of *T. denticola*-dependent MMP transcription. *T. denticola* produces dentilisin, which promotes tissue-destructive cellular processes through a TLR2/MyD88/Sp1-dependent mechanism (Ganther et al., 2021). Dentilisin is a surface protease, that degrades host proteins such as transferrin, fibrinogen, gelatin, fibronectin, and laminin. It exhibits a cytopathic effect on human epithelial cells and is involved in fibrinogen binding (Kokubu et al., 2021). Additionally, it mediates adherence to fibronectin and plays a role in immunomodulation (Arai et al., 2020). It has been described that *T. denticola* can infiltrate the brain and directly influence nerve cells, potentially contributing to the deposition of Aβ and the subsequent pathological progression of Alzheimer’s disease (Su et al., 2021).

### Aggregatibacter actinomycetemcomitans

*A. actinomycetemcomitans* is a non-motile, facultative anaerobic or capnophilic, Gram-negative coccobacilli bacterium. It grows well in microaerophilic conditions of 5–10% CO_2_ and plays a primary role in the development of PD. It is also linked to AgPD, which can lead to premature tooth loss in adolescents. Its involvement in gingival dysbiosis during the early stages of PD is crucial. *A. actinomycetemcomitans* harbors a 14-gene operon, known as the widespread colonization island, housing the tight adherence (*tad*) genes. This operon is associated with strong biofilm formation capabilities and robust attachment to enamel-like substrates *in vitro* (Velusamy et al., 2019; Fine et al., 2020; Isola et al., 2020). *A. actinomycetemcomitans* colonizes the oral cavity by adhering to gingival epithelial cells through both fimbria andnon-fimbria- dependent adhesins, including Aae. Additionally, it contributes to the formation of dental biofilms (Ishikawa et al., 2021). Both fimbriae and nonfimbrial adhesins can either specifically target a particular substrate or show affinity to several different cellular targets. In addition to their role in biofilm formation, they play an important role in the invasion of host cells (Danforth et al., 2021). *A. actinomycetemcomitans* can damage human white blood cells by producing exotoxins, including leukotoxin (LtxA) and the cytolethal distending toxin (CDT) (Khzam et al., 2024). CDT is a genotoxin that disrupts the host cell cycle, kills host cells and contributes to tissue destruction. CDT consists of three subunits including CdtA, CdtB, and CdtC. The active subunit, CdtB, possesses DNase activity and infiltrates host cells, causing DNA damage. This mechanism aligns with its role in the pathogenesis of PD (DiRienzo, 2014). LtxA, an important virulence determinant, specifically targets leukocytes and triggers rapid caspase-1 activation, leading to substantial IL-1β secretion in human monocytes and macrophages. The NLRP and AIM2 inflammasomes, along with ROS and cathepsin B, likely participate in this process (Cheng et al., 2020). LtxA also triggers PMN degranulation and the extracellular release of proteolytic enzymes such as elastase and MMPs, which cause host cell death by inhibiting cell proliferation and enhancing the expression of receptor activator of RANKL, a key factor in osteoclastogenesis (Åberg et al., 2015). *A. actinomycetemcomitans* is the only pathogen associated with CPD capable of synthesizing LtxA. This toxin mimics the citrullinated pattern observed in joints affected by rheumatoid arthritis (RA) by activating *P. gingivalis* peptidylarginine deiminase and inducing hypercitrullination in neutrophils through pore formation (Huang and Ni, 2024).

*A. actinomycetemcomitans* employs various strategies to survive within multispecies biofilms. One such strategy is its ability to regulate catalase production, which helps break down hydrogen peroxide (H_2_O_2_) produced by other organisms such as Streptococci. Another strategy involves the production of dispersin B, which aids in releasing of *A. actinomycetemcomitans* cells from the biofilm matrix (Stacy et al., 2014). Six distinct serotypes of *A. actinomycetemcomitans* have been characterized based on LPS antigenicity. Among these, serotype C is the most commonly isolated from patients with PD (Shahoumi et al., 2023). *A. actinomycetemcomitans* employs several strategies to evade the host defense, including hindering leukocyte chemotaxis, producing immunosuppressive factors, secreting IgG-cleaving proteases, and synthesizing Fc-binding proteins (Bapat et al., 2022). In addition to PD, *A. actinomycetemcomitans* may contribute to the development and progression of systemic conditions, including infective endocarditis, bacteremia, meningitis, and skin infections (Nørskov-Lauritsen et al., 2019).

### Fusobacterium nucleatum

*Fusobacterium nucleatum* is an obligate anaerobic, Gram-negative, rod-shape bacterium belongs to the genus Fusobacterium. It is named for its slender appearance and spindle-like tips at both ends. *F. nucleatum* often found as a resident in various parts of the human body microbiota, especially in the oral cavity (Ghosh et al., 2024). It is present in small quantities in healthy subgingival dental biofilm but becomes more abundant in periodontal pockets (Jung et al., 2017). *F. nucleatum* plays a crucial structural role in connecting early and late colonizers of dental plaque. Recent studies have shown that the cross-feeding and trophic interactions between *F. nucleatum* and early-colonizing microorganisms can impact biofilm formation and the prolifertion of later colonizing pathogens, such as *P. gingivalis* (Baker et al., 2024). *F. nucleatum* pathogenesis is primarily associated with several virulence determinants including adhesins on its surface, such as RadD, Aid1, and FomA, which can co-aggregate bacteria to facilitate biofilm formation (Chen et al., 2022a). Fusobacterium adhesin A (FadA) is the only adhesin expressed by *F. nucleatum* that binds to host cells. FadA exists in two forms: non-secreted, intact pre-FadA, and secreted, mature FadA (mFadA). Through its interactions with host cells, FadA induces tumorigenic responses and facilitates bacterial invasion (Groeger et al., 2022). *F. nucleatum* can invade various cell lines, including oral, colonic, placental epithelial cells, immune cells, and keratinocytes. Once internalized, *F. nucleatum* induces the expression of specific cytokines, regulates cell proliferation and apoptosis, and other biological behaviors, ultimately leading to epithelial cell dysfunction and destruction of periodontal tissue (Zhang et al., 2022). LPS is another virulence factor of *F. nucleatum* that plays a crucial role in the production of epithelial cytokines by activating TLR-4 (Engevik et al., 2021). *F. nucleatum* secretes a serine protease that not only meets its nutritional needs but also damages host tissues. This enzyme degrades ESM proteins, leading to the breakdown of periodontal connective tissues, immunoglobulins, and complement proteins in the host immune system. Specifically, it cleaves the α-chain of IgA, supporting *F. nucleatum* in evading the host’s immune system (De Andrade et al., 2019). Butyric acid, a short-chain fatty acid (SCFA) produced by *F. nucleatum*, can influence the damage and healing of periodontal tissues. Elevated levels of butyric acid can increase ROS formation in osteoblasts, which in turn stimulates the secretion of 8-isoprostaglandin and MMP-2. This process leads to bone destruction and impairs bone repair (Chen et al., 2022a). *P. gingivalis* is sensitive to acidic conditions. However, *F. nucleatum* can ferment glutamate and aspartate to produce ammonia, creating a more neutral environment that supports the colonization of *P. gingivalis* (McIlvanna et al., 2021). In addition to PD, the potential role of *F. nucleatum* has been described in a variety of diseases, such as, endodontic infections, gingivitis, tonsillitis, head and neck tumor, appendicitis, inflammatory bowel diseases, gastrointestinal tumor, endocarditis, atherosclerosis, respiratory tract infections, abscesses, bone infections, and adverse pregnancy outcomes (including preterm labor, stillbirth, and chorioamnionitis), as well as urinary tract infections (Brennan and Garrett, 2019).

### *Prevotella* spp.

*Prevotella* are Gram-negative, obligate anaerobes typically found in the human vaginal microbiota, gastrointestinal tract, respiratory tract, and oral cavity (Larsen, 2017). These species are characterized as non-motile, non-spore-forming rods, with colony colors ranging from shiny white to black (George et al., 2024). Periodontal pathogens from the Prevotella genus including *P. intermedia*, *P. melaninogenica*, *P. nigrescens*, *P. denticola*, *P. corporis*, and *P. disiens* have been identified (Arora et al., 2014). *P. intermedia*, a black-pigmented anaerobic rod that possesses various virulence factors such as adhesion, hemolysin, hemagglutinin, proteolytic and hydrolytic enzymes. These factors allow *P. intermedia* to establish itself in the oral cavity, evade and modulate the hosts immune defenses, and cause tissue damage (Eley and Cox, 2003). Additionally, *P. intermedia* has been shown to induce the expression of pro-MMP-2 and pro-MMP-9 in fetal mouse osteoblasts (Pelt et al., 2002).

Additionally, it has been shown that *P. intermedia* can stimulate the production of MMP-9 in human periodontal ligament (hPDL) cells (Guan et al., 2008). It upregulates the mRNA expression and protein secretion of MMP-1 and MMP-8 through MAPK signaling pathways and PGE2 synthesis in these cells. This suggests that *P. intermedia* may play a role in the degradation of periodontal connective tissue and bone matrix during CP by increasing the expression of multiple MMPs (Guan et al., 2009).

### Campylobacter gracilis

*Campylobacter gracilis*, formerly known as *Bacteroides gracilis*, was first identified in patients with gingivitis and PD (Tanner et al., 1981). *C. gracilis* is an anaerobic Gram-negative rod shaped bacterium that is non-motile and non-spore-forming. The type strain of *C. gracilis* type strain possesses multiple potential virulence factors, such as hemagglutinins, toxins, immunity proteins, and other predicted factors. Furthermore, the genomic island containing zonula occludens toxin (zot) has been found in this strain (Miller and Yee, 2015). It primarily inhabits the gingival sulcus and is known for its contribution to the progression of PD. While its involvement in PD is well established, systemic infections caused by *C. gracilis* are extremely rare, with only a few cases reported in the medical literature (Arakawa et al., 2024). *C. gracilis* has been linked to various diseases, such as Crohn’s disease in children and ulcerative colitis in adults (Man, 2011).

### Veillonella parvula

*Veillonella parvula* is an anaerobic, small and nonmotile Gram-negative coccus that is part of the human normal flora. Although *Veillonella* spp. are generally considered commensal microorganisms, they have occasionally been implicated in infections, particularly in immunocompromised individuals (Gouze et al., 2019). *Veillonella* spp. are typically present in biofilms and often co-aggregate with lactic acid bacteria (Rojas-Tapias et al., 2022). *V. parvula* is an important early colonizer of dental plaque, aiding in the biofilm’s formation. It promotes the growth of multiple species and plays a crucial role in the community’s metabolism by consuming lactic acid (Béchon et al., 2020). Despite its role in biofilms, *V. parvula* is also known as an opportunistic pathogen associated with various infections such as osteomyelitis, endocarditis, spondylodiscitis, abscesses, and systemic infections (Hirai et al., 2016; Li et al., 2017; Stefani et al., 2017; Hyo et al., 2019; Wellens et al., 2019). *V. parvula* does not ferment carbohydrates instead, it relies on lactate produced by Streptococci as its carbon source for growth. In dual-species biofilms, *V. parvula* has been shown to enhance the growth and extracellular polysaccharide (EPS) synthesis of *S. mutans* (Liu et al., 2020). Delwiche et al (Delwiche et al., 1985). described that Veillonella spp. generate significant amounts of LPS. Additionally, *V. parvula* LPS induces cytokine production and activates p38 MAPK dependent on TLR-4 (Matera et al., 2009). These characteristics of Veillonella SPP. are believed to complicate the treatment of *V. parvula*-associated PD (Mashima et al., 2016). Veillonella are regarded as a bridging species because of its capacity to modify the microbiome environment. The catalase produced by *V. parvula* can provide a more desirable low redox potential for oxygen-sensitive anaerobes, thereby enhancing the potential of pathogenic microorganisms to thrive in the oral microbiome (Garcia, 2022).

## Treatment of periodontitis

The successful treatment of an infection relies on accurately diagnosing of the pathogens contributing to its etiopathogenesis. Diagnosing an infection can be challenging, especially in patients with a polymicrobial infection in organs are naturally colonized by bacteria, like the skin, gastrointestinal tract and oral cavity. Methodological problems in detecting the complex subgingival microbial population, which is heavily colonized by numerous species of strict and facultative anaerobes and fastidious bacteria, have significantly hindered the accurate diagnosis and antibiotic therapy of PD (Socransky and Haffajee, 1994). The treatment of PD should be started as soon as possible. Mild to moderate cases of PD are typically treated using nonsurgical procedures, such as supplementary antimicrobial agents, dental scaling, and root planning (Slots, 2012; Albandar, 2014). Nonsurgical treatments are typically not enough for sever PD. Surgical procedures are necessary to reduce pocket depth and establish anatomical contours at the periodontal junction (Yang et al., 2021). Systemic antimicrobial drugs are administered as monotherapy or in combination therapy. However, it is highly recommended to combine antibiotics with non-surgical periodontal therapy to achieve optimal clinical outcomes (Souza et al., 2020). The most common antibiotic regimens for PD include β-lactams, tetracyclines, quinolones and metronidazole (Keestra et al., 2015). The combination of metronidazole and amoxicillin is the most frequently used antimicrobial therapy for PD (Elashiry et al., 2021). This combination has been shown to have synergic effects, reducing the necessary levels of both antibiotics for biological effects (Mugri, 2022). A 7-day regimen of 500/500 mg or 500/400 mg of amoxicillin and metronidazole is recommended for PD (McGowan et al., 2018).

Azithromycin is a macrolide agent generally administer as an alternative in case of penicillin-allergy. The suggested regimen for azithromycin is 500 mg/day for 3 days and it an assists in non-surgical procedures and mechanical plaque elimination in the treatment of PD (Jones and Hoyle, 2022). Macrolides should not be combine with clindamycin, due to their similar cellular target and antagonistic effects (Flynn, 2019).

Clindamycin is a lincosamide agent that has a bacteriostatic effect on anaerobic bacteria and is highly effective against mixed infections caused by both anaerobic and aerobic bacteria. It has favorable pharmacokinetics and has become a commonly prescribed alternative for oral infections in patients with penicillin allergy. The common oral dosage of clindamycin is 300 mg every 6 h (Jeske, 2024). Clindamycin is the only antimicrobial drug that reduces the attachment of bacteria to epithelial cells on the mucosal surface by decreasing the expression of microbial factors. It also decreases the production of proinflammatory cytokines, such as TNF-α and IL-1β, which can lead to additional destruction of periodontal tissues, when overexpressed by microorganisms and neighboring cells. Therefore, the decreased release of TNF-α and chemokine CXCL-1 are additional effects of clindamycin, which help to suppress the inflammatory condition such as PD (Luchian et al., 2021).

Tetracycline derivatives are generally administer as supportive agents in PD antimicrobial therapy (Mohammad et al., 2022). Tetracycline has a long shelf-life, which preserve its antibacterial effects for an extended period, and is slowly released from the tooth surface (Kafle et al., 2018).

Tetracycline also possesses a surprising capacity to prevent host-derived MMP effects and connective tissue damage decreases the release of inflammatory mediators, and lead to the concept of host-modulation therapy in the treatment of PD. Upregulated collagen synthesis, osteoblast activity, and bone formation are non-antimicrobial properties of tetracycline, making it an appropriate option for PD treatment (Golub and Lee, 2020).

Ciprofloxacin is a broad-spectrum antibiotic that is effective against various pathogens -associated PD, including *A. actinomycetemcomitans*. It efficiently penetrated the infected periodontal tissues and can reach higher levels in the crevicular fluid compared to the blood.

Because periodontal infections are commonly polymicrobial and caused by a variety of periodontal pathogens, a combination of antibiotics is often used to treat AgPD (Bidault et al., 2007).

Moxifloxacin, another quinolone, has shown significant antimicrobial effects on PD-associated pathogens *in vitro* and in a clinical study involving AgPD cases. It has been shown that daily 400 mg of moxifloxacin, used in conjunction with one-stage full-mouth cleaning and root planning improved clinical outcomes compared to mechanical treatment alone in AgPD cases (Ardila et al., 2015). However, moxifloxacin is not typically administered as the first-line drug due to its high cost, and is usually reserved for case where first-line antimicrobials and surgical procedures have been unsuccessful (Holmes and Pellecchia, 2016).

## Alternative treatments of periodontitis: over antibiotics and surgical procedures

Understanding the role of oral microbiota dysbiosis in the initiation and progression of PD is crucial for devising effective therapeutic strategies aimed at reducing bacterial load and restoring microbiological balance (Sachelarie et al., 2025). Conventional treatments for periodontitis often don’t completely eliminate harmful pathogens (Matsubara et al., 2016). However, when executed meticulously, these treatments can promote a healthier oral environment by modifying the composition and population of the microbial community and aiding in the maturation of the host immune response (Fragkioudakis et al., 2021). The primary methods for treating periodontitis include controlling biofilms, mechanically removing plaque, and using antibiotic therapy (Slots, 2017). In recent years, the main goal of these treatments has evolved to focus on restoring homeostasis within the oral microbiota. However, bacterial strains involved in PDs are increasingly becoming resistant to antibiotics, posing a significant challenge to conventional treatments (Haque et al., 2022). Consequently, there has been a growing focus on complementary approaches to enhance traditional mechanical treatments. Among these, probiotics have gained attention from researchers and clinicians for their potential to help restore microbial balance. Probiotics produce compounds such as lactic acid, hydrogen peroxide, and bacteriocins, which can reduce pathogenic bacterial biofilms and decrease levels of pro-inflammatory factors like cytokines, collagenases, elastases, and prostaglandin E2. These probiotic bacteria then adhere more firmly to the oral cavity’s surface, preventing new pathogens from colonizing. Through this process, bacterial aggregation and co-aggregation occur, leading to a new microbial balance that forms a healthy biofilm (Mishra et al., 2020). The incorporation of probiotics in periodontal treatment represents a promising innovation with the potential to significantly enhance long-term clinical results. While phage therapy has demonstrated efficacy against numerous infections, its application in the treatment of oral diseases remains underexplored.

Bacteriophage therapy offers a novel and promising alternative method of treatment (Shlezinger et al., 2017). Bacteriophages, also known as phages, are viruses that infect bacteria by entering the host cell and initiating a cycle of phage production. They play a crucial role in the infectious cycle of lytic phages, ultimately leading to the lysis or death of the bacterial host (Zhu et al., 2025). Phages, which are highly selective, non-toxic, self-replicating, and capable of infiltrating biofilms, offer a novel alternative to traditional biofilm prevention techniques (Szafrański et al., 2017). Their ability to combat dental plaque is highlighted by their small size, allowing them to penetrate biofilm layers with high efficiency. Phage therapy could become a crucial treatment option for root canal infections that are resistant to conventional endodontic methods and has shown effectiveness against root canal infections caused by *Enterococcus faecalis*, a foodborne pathogen associated with various diseases and potentially implicated in periodontal health (Łasica et al., 2024; Hosseini Hooshiar et al., 2024).

Over the past century, lasers have been used to treat variety of diseases, including periodontitis. A laser device generates electromagnetic radiation at a specific wavelength and a low-intensity beam, which has significant effects on tissues. The use of lasers is considered beneficial for treating a range of infectious and inflammatory conditions (Isola et al., 2021). Several studies have shown that lasers can promote periodontal wound healing and regeneration by effectively removing and decontaminating diseased tissues, as well as by modulating or activating cell metabolism in the surrounding tissues. In the past decade, low-intensity diode lasers have been used in combination with photosensitizers to activate topical photosensitizing agents such as antimicrobial photodynamic therapy (aPDT), to help reduce or eliminate periodontopathogen bacteria as a complement to mechanical debridement in patients with periodontitis (Chambrone et al., 2018). Photodynamic therapy (PDT) is a non-thermal photochemical reaction that requires the simultaneous presence of visible light, oxygen, and a dye or photosensitizer (PS). Various PS have been studied for their ability to bind to bacteria and effectively produce reactive oxygen species (ROS) when exposed to light. These ROS are generated through type I or II mechanisms and can deactivate various types of bacterial cells (Sperandio F et al., 2013). The most common laser applications for periodontal therapy include diode, carbon dioxide (CO2), and neodymium-doped lasers. These wavelengths can be used in conjunction with mechanical non-surgical instrumentation to debride connective tissue and epithelium within periodontal pockets, inactivate bacteria, and ablate subgingival calculus (Salvi et al., 2020).

Various local and systemic approaches have been employed to effectively treat periodontitis. Currently, controlled local drug delivery is more favored compared to systemic methods. This is because it focuses on enhancing therapeutic outcomes by achieving factors such as site-specific delivery, low dose requirements, bypassing first-pass metabolism, reducing gastrointestinal side effects, and decreasing dosing frequency (Joshi et al., 2016). Selecting the right antimicrobial agent with the appropriate route of administration is crucial for successful periodontal therapy. Local drug delivery systems (LDDS) such as irrigating systems, fibers, gels, strips, films, microparticles, nanoparticles, and low-dose antimicrobial agents are available to deliver antimicrobial agents to sub-gingival diseased sites with minimal or no side effects on other body sites (Rajeshwari et al., 2019).

## Antimicrobial resistance

Several studies have revealed that levels of resistance to certain antibiotics increased have in microbial agents involved PD. The most significant factor contributing to the rise in antimicrobial resistance is the misuse or overuse of these agents. Resistance to metronidazole in PD- associated pathogens has been reported in several studies (Mínguez et al., 2019). Higher levels of metronidazole resistance have been reported in *A. actinomycetemcomitans* compared to *P. gingivalis.* The resistance frequency of *P. gingivalis* strains from Colombia to amoxicillin, azithromycin, and metronidazole has been reported as 24.6%, 21.3% and 24.6%, respectively (Ardila and Bedoya-García, 2020). However, some studies have described the sensitivity of all *P. gingivalis* strains to all or most tested antimicrobial agents (Mínguez et al., 2019; Rams et al., 2023).

Some β-lactamase-producing pathogens may contribute to refractory PD. Since PDs commonly are polymicrobial, the presence of β-lactamase-producing pathogens may prevent nonproducing organisms in subgingival plaques from being affected by β-lactam agents, leading to treatment failure or disease recurrence (Handal et al., 2004). A significant increase in resistance to clindamycin and amoxicillin has been reported over a 20-year period among *P. gingivalis* isolated from severe PD patients in the United States (Rams et al., 2023).

Choosing the appropriate antibiotics for PD is a complex and challenging due to the presence of over 700 bacterial species in the oral cavity (Radaic and Kapila, 2021). On the other hand, selecting an antimicrobial agent for PD treatment based on microbiological analysis and antibiotic susceptibility pattern is practically impossible. There are few guidelines on how to determine and interpret antimicrobial resistance patterns of anaerobic oral bacteria because there is inadequate information on the relationship between minimal inhibitory concentrations (MIC) of antibiotics, actual local (oral) levels *in vivo* (PK/PD data) and clinical outcomes (Jepsen et al., 2021). On the other hand, most of the bacteria that cause PD are fastidious, requiring complex culture media and nutritional supplements for *in vitro* growth, as well as a long incubation period.

It is well known that antibiotic resistance varies between countries based on their usage patterns and levels of antibiotic administration in general clinical settings (Van Winkelhoff et al., 2005). Antimicrobial drugs are prescribed empirically based on the periodic antibiotic sensitivity patterns reported by national researches (Luan et al., 2023). Undoubtedly, prospective clinical and surveillance studies, as well as monitoring the resistance levels to antibiotics will be effective in the optimal usage of antibiotics and control of drug-resistant strains.

## Conclusion

The microbial-biofilm formation and dental plaque is the initial stage of PD. The extended presence of plaque on the teeth’s surface causes it to migrate into the surrounding periodontal tissues. This spread triggers the infiltration of host immune cells, leading to inflammation of the gingival tissue and ultimately bone destruction. The damage and injuries caused by PD result from immune system mediators and virulence factors produced by pathogenic bacteria. These effects are primarily due to interactions, co-aggregation, and metabolic dependencies among the pathogens. Therefore, prevention of dental plaques and antimicrobial therapy can have a significant impact on prevention and treatment of PD. The prospective clinical and surveillance studies, as well as monitoring the resistance levels to antibiotics will be effective in the optimal usage of antibiotics and control of drug-resistant pathogens caused PD. Novel approaches like NGS and OMICS-based methods can be used to more accurately study the microbiology and pathology of PD. Understanding the role and effects of bacterial secondary metabolites, such as short-chain fatty acids, produced by the microbiome in the prognosis and pathogenesis of PD provides valuable data the prevention and the development of new therapeutic and diagnostic methods. Because PD is polymicrobial and there is increasing antibiotic resistance among its pathogens, there is an urgent need to update laboratory and clinical guidelines and assess precise and reliable methods for detecting resistant strains.
